# Specific Depletion of Ly6C^hi^ Inflammatory Monocytes Prevents Immunopathology in Experimental Cerebral Malaria

**DOI:** 10.1371/journal.pone.0124080

**Published:** 2015-04-17

**Authors:** Beatrix Schumak, Katrin Klocke, Janina M. Kuepper, Aindrila Biswas, Andrea Djie-Maletz, Andreas Limmer, Nico van Rooijen, Matthias Mack, Achim Hoerauf, Ildiko Rita Dunay

**Affiliations:** 1 Institute of Medical Microbiology, Immunology and Parasitology, University of Bonn, Bonn, Germany; 2 Institute of Medical Microbiology, University of Magdeburg, Magdeburg, Germany; 3 Department of Neurosurgery, University of Freiburg, Freiburg, Germany; 4 Institutes of Molecular Medicine and Experimental Immunology, University of Bonn, Bonn, Germany; 5 VUMC Department of Molecular Cell Biology, Faculty of Medicine Vrije Universiteit, Amsterdam, The Netherlands; 6 Department of Internal Medicine II, University Hospital Regensburg, Regensburg, Germany; Université Pierre et Marie Curie, FRANCE

## Abstract

*Plasmodium berghei ANKA* (PbA) infection of C57BL/6 mice leads to experimental cerebral malaria (ECM) that is commonly associated with serious T cell mediated damage. In other parasitic infection models, inflammatory monocytes have been shown to regulate Th1 responses but their role in ECM remains poorly defined, whereas neutrophils are reported to contribute to ECM immune pathology. Making use of the recent development of specific monoclonal antibodies (mAb), we depleted *in vivo* Ly6C^hi^ inflammatory monocytes (by anti-CCR2), Ly6G^+^ neutrophils (by anti-Ly6G) or both cell types (by anti-Gr1) during infection with Ovalbumin-transgenic PbA parasites (PbTg). Notably, the application of anti-Gr1 or anti-CCR2 but not anti-Ly6G antibodies into PbTg-infected mice prevented ECM development. In addition, depletion of Ly6C^hi^ inflammatory monocytes but not neutrophils led to decreased IFNγ levels and IFNγ^+^CD8^+^ T effector cells in the brain. Importantly, anti-CCR2 mAb injection did not prevent the generation of PbTg-specific T cell responses in the periphery, whereas anti-Gr1 mAb injection strongly diminished T cell frequencies and CTL responses. In conclusion, the specific depletion of Ly6C^hi^ inflammatory monocytes attenuated brain inflammation and immune cell recruitment to the CNS, which prevented ECM following *Plasmodium* infection, pointing out a substantial role of Ly6C^+^ monocytes in ECM inflammatory processes.

## Introduction

Malaria remains one of the most serious infectious diseases affecting 10% of the world's population. Although infections are endemic in over 100 countries, 90% of the deaths, most of which affect children, occur in sub-Saharan Africa and South East Asia [1, 2]. Malaria is elicited by various species of the protozoan parasite from the genus *Plasmodium* and is transmitted to humans through the bite of female *Anopheles* mosquitoes. *P*. *falciparum* is the most virulent of the five *Plasmodium* species that cause disease in humans. Amongst the serious pathological complications, cerebral malaria (CM) remains the greatest life-threatening risk. CM is a fatal neurological syndrome with multi-factorial, complex developmental stages and symptoms. It is generally acknowledged that CM results from immune-mediated pathology due to overwhelming inflammatory processes and parasite sequestration [3]. Infections in C57BL/6 mice with *Plasmodium berghei ANKA* (PbA) infected red blood cells induce lethal experimental CM (ECM) [4, 5]. The resulting cerebral pathology in PbA-infected mice is induced by pro-inflammatory immune responses of CD8^+^ T cells and subsequent IFNγ production [6–8]. However, it remains unclear how such strong immune responses are induced or regulated and the exact contribution of phagocytic cells in ECM is incompletely understood.

In the present study we addressed the question about the contribution of inflammatory monocytes in ECM development. In our previous studies, we demonstrated that a primary function of inflammatory monocytes (Gr1^+^Ly6C^hi^CCR2^+^CX_3_CR1^lo^), a subset of mononuclear cells, was to drive strong Th1 responses within the host in the murine model of *Toxoplasmosis* [9, 10]. Furthermore, Ly6C^hi^ monocytes were recruited to the site of infection and there, contributed to disease control via secretion of anti-microbial molecules [9, 11, 12]. Such Ly6C^hi^CCR2^+^ monocytes emerge from the bone marrow and populate non-lymphoid tissues [9, 13, 14]. They contribute to orchestrate memory CD8^+^ T cell and NK cell activation via the production of interleukin 18 and interleukin 15 [15]. In the absence of the CCR2 receptor, monocytes are unable to exit the bone marrow and in accordance, CCR2^-/-^ mice display increased susceptibility to Listeria *and* Toxoplasma *infections* [9, 14]. In contrast to inflammatory Gr1^+^Ly6C^hi^CCR2^+^CX3CR1^lo^ cells, the other major subset of monocytes, Gr1^−^Ly6C^-^CCR2^−^CX_3_CR1^hi^, establish residency in the periphery where they perform important surveillance actions [16]. Similar subsets of monocytes have been described in humans; CD14^+^CD16^−^ vs. CD14^lo^CD16^+^ cells which represent inflammatory and surveillance populations, respectively [17, 18]. In addition to monocytes, Gr1 is expressed on neutrophils and the most common antibody used to define this receptor, RB6, recognizes both Ly6C and Ly6G isoforms [11, 19]. Recently, monoclonal antibodies (mAbs) detecting distinct Ly6 isoforms have been developed allowing neutrophils (CD11b^+^F4/80^−^Ly6G^hi^Ly6C^int^Gr1^hi^) to be readily distinguished from inflammatory monocytes (CD11b^+^F4/80^+^Ly6C^hi^Ly6G^––^CCR2^+^Gr1^int^) and selectively depleted [11, 19, 20]. With regards to the role of Gr1^+^ cells during malaria infection, previous depletion studies conducted by Chen *et al*., using the anti-Gr1 antibody (clone RB6) in PbA infected mice, concluded an important role for neutrophils in developing ECM pathology [21]. However, recent reports demonstrated broad elimination of Gr-1 positive cells including monocytes and T cells upon anti-Gr1 injection, thus questioning the validity of this clone. Here, we describe the impact of Ly6C^hi^ inflammatory monocytes on ECM pathology during experimental *Plasmodium* infection using the new selectively depleting antibodies anti-CCR2 and anti-Ly6G to analyse the impact of Ly6C^hi^ inflammatory monocytes *versus* neutrophils in the development of ECM.

## Results

### Depletion of phagocytic cells prevents ECM in PbTg-infected mice

**Fig 1:**
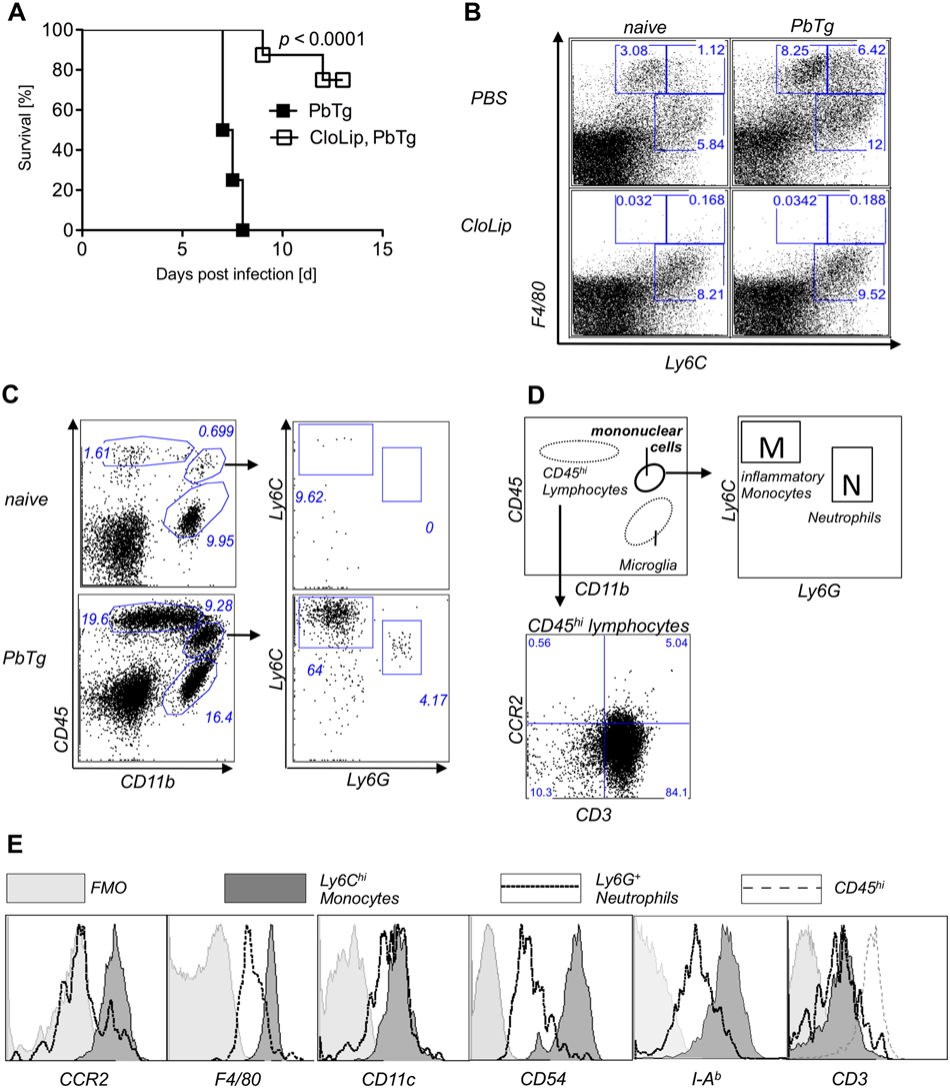
Depletion of Ly6C+F4/80+ cells, which are present in brains of ECM positive mice, prevents ECM. (A) Survival of PbTg-infected C57BL/6 mice. 24 hr before inoculation with iRBC (5*10^4^) of PbTg, indicated C57BL/6 mice received 200μl CloLip i.v.. Graph shows survival rates of mice p.i. (n = 10 mice per group) and represents 1 of 2 independent infection studies. Statistical analysis was performed using log-rank test. (B) Flow cytometric analysis of Ly6C^+^F4/80^+^ cells in spleens of non-depleted mice (upper row) and CloLip depleted mice (lower row), which were either naïve (uninfected) (left panel) or PbTg infected (right panel; 24 hours p.i.). Dot plots show representative stainings from 1 of 3 independent depletion studies in 3 mice per group. (C) Emergence of peripheral lymphocytes and mononuclear cells in brains of PbTg infected mice. Flow cytometric analyses show representative plots of naïve and PbTg infected mice, day 6 p.i.. Expression of CD45 and CD11b were used to discriminate lymphocytes (CD45^hi^ CD11b^-^) and mononuclear cells (CD45^+^CD11b^+^) from brain-resident microglia (CD45^-^CD11b^+^). (D) Scheme for discrimination of cell populations and gating of Ly6C^hi^Ly6G^-^ inflammatory monocytes [M] versus neutrophils (Ly6C^int^Ly6G^+^) [N] out of mononuclear cells as shown in data set of (B). CD45 hi cells were identified as CD3^+^ T cells (lower panel). (E) Ly6C^hi^Ly6G^neg^ inflammatory monocytes but not Ly6G^+^ neutrophils from the brain of PbTg infected mice express CCR2, F4/80, CD54 and I-A^b^. Flow cytometric data of brains isolated from PbTg infected animals, which were gated as depicted in (C). FMO = fluorescence minus one are shown as control. Representative data from 1 of 3 independent experiments are shown.

Although ECM in PbA infected C57BL/6 mice is predominantly mediated by CD8^+^ T cells and IFNγ [6–8], the exact contribution of responding phagocytic cell subpopulations in developing such Th1 responses remains insufficiently defined. Therefore, we examined the participation of phagocytic cells in the development of ECM using a transgenic strain of *Plasmodium berghei ANKA* that expresses ovalbumin (PbTg) [22]. Initially, groups of C57BL/6 mice were intravenously injected with clodronate liposomes (CloLip) that result in the depletion of all cells possessing phagocytic activity [23] and then infected with PbTg. CloLip administration protected mice from developing lethal ECM and resulted in an 80% survival rate (Fig 1A). Parasitemia levels did not differ significantly between the infected groups (Supplementary information S1 Fig). When compared to naïve mice, flow cytometric analysis of splenocytes from PbTg-infected C57BL/6 mice revealed increased frequencies of phagocytic cells, in particular more Ly6C^hi^F4/80^+^ monocytes (6.42% vs. 1.12%), Ly6C^int^F4/80^+^ monocytes (8.25% vs. 3.08%) and Ly6C^+^F4/80^-^ neutrophils (12% vs 5.84%) (Fig 1B, upper row). As expected, F4/80^+^ cells were depleted upon injection of CloLip, in both PbTg infected C57BL/6 mice and uninfected control mice (Fig 1B lower panel), in comparison to non-depleted mice, that received PBS-loaded liposomes (Fig 1B, upper panel). Monocytes express high levels of both Ly6C and F4/80 and were depleted upon protective CloLip administration. On the other hand, there are data for a detrimental role of neutrophils in *P*. *berghei ANKA* induced ECM [21]. Therefore, we investigated the role of monocytes versus neutrophils in the ECM model. First, we evaluated the composition of cellular infiltrates in brains of PbTg-infected mice at day 6 post infection (p.i.) and sought for the presence of lymphocytes, inflammatory monocytes (defined by Ly6C^hi^Ly6G^-^, box M) and neutrophils (defined by Ly6C^int^Ly6G^+^, box N) (see gating scheme in Fig 1C and 1D). We observed that in the brains of PbTg-infected C57BL/6 mice with ECM, compared to naïve animals, there was a strong influx of CD45^hi^ leukocytes, which were positive for CD3 (Fig 1D, lower panel), and CD45^+^CD11b^+^ mononuclear cells from the periphery that could be discriminated from CD45^-^CD11b^+^ microglia. In brains of PbTg-infected mice, but not in brains of naïve mice were both Ly6C^hi^Ly6G^-^ cells (inflammatory monocytes) and CD11b^+^Ly6G^+^Ly6C^int^ cells (neutrophils) among the CD45^+^CD11b^+^ mononuclear cells present (Fig 1C lower panel right). The inflammatory monocytes from brains of PbTg infected mice also showed strong expression of CCR2 and F4/80, MHC class II, and CD54, in contrast to Ly6G^+^ neutrophils, which showed either intermediate expression of those surface molecules or were devoid of these surface molecules (Fig 1E). Both mononuclear cell populations showed intermediate expression of CD11c, but were negative for CD3 (Fig 1E).

### Early depletion of CD11b^+^Ly6C^hi^ inflammatory monocytes in *Plasmodium* infection protects mice from ECM

**Fig 2:**
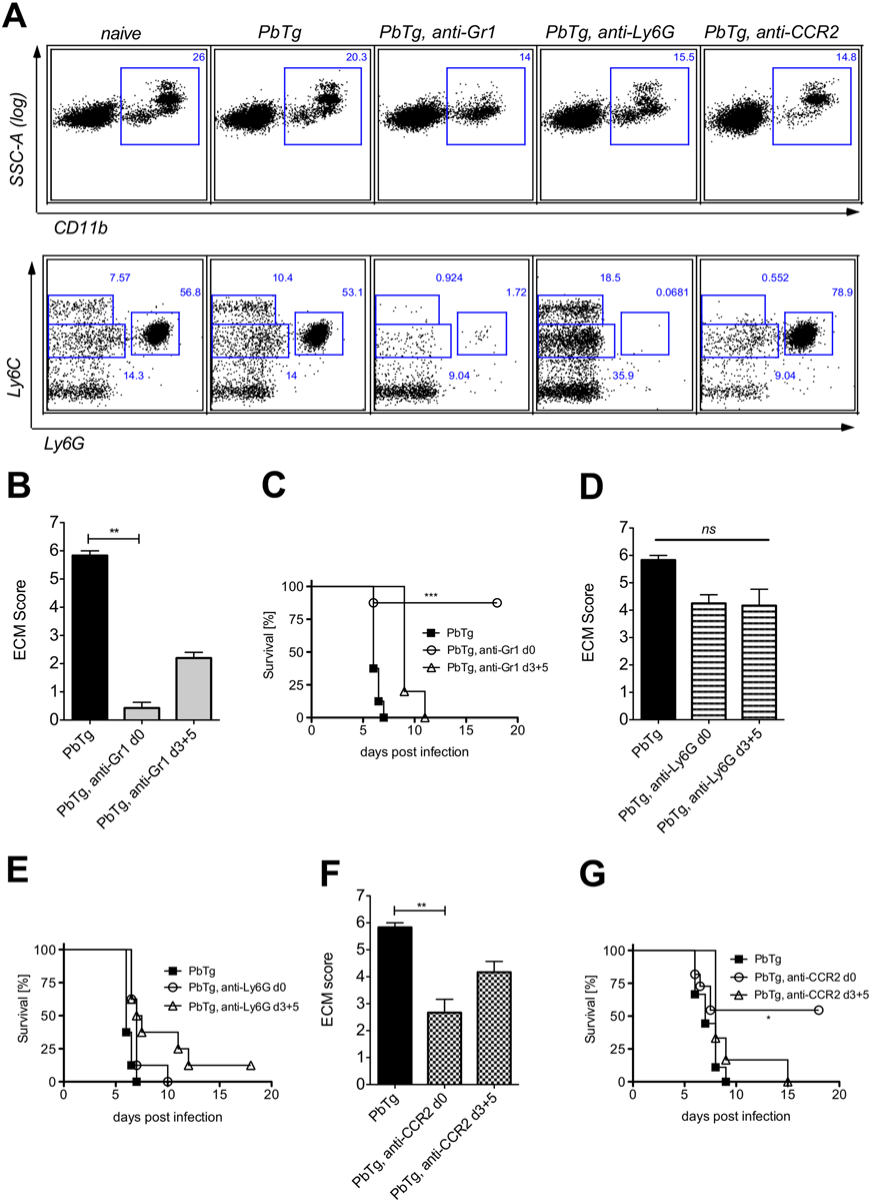
Early depletion of inflammatory monocytes protects PbTg infected mice against arising ECM. (A) Effective depletion of inflammatory monocytes (Ly6C^hi^Ly6G^-^) or neutrophils (Ly6C^int^Ly6G^+^) in the blood of PbTg-infected C57BL/6 mice upon treatment with anti-Gr1 (middle), anti-Ly6G (right), or anti-CCR2 (far right) monoclonal antibodies, on d+1 post depletion. CD11b^+^ leukocytes from the blood (upper row) were gated (squares) and analysed for the presence of Ly6C^+^Ly6G^-^ monocytes and Ly6^int^Ly6G^+^neutrophils (lower row) The data of the lower row correspond to data of the upper row. n = 4 mice per group. Flow cytometric analyses show a representative data plot for each group from 1 of 3 independent experiments. (B-G) Groups of PbTg-infected C57BL/6 mice were injected i.p. with anti-Gr1 mAb (B and C), anti-Ly6G mAb (D and E) or anti-CCR2 mAb (F and G) on the first day of infection (d0) or on days 3 and 5 p.i. (d3+5). Scores of cerebral pathology were determined on day 6 p.i. and bar graphs in B, D and F depict the mean and SEM of ECM score in individual mice (n = 8 mice per group). Statistical analysis was performed using Kruskal-Wallis test and Dunn’s Post test (** *p*<0.01). Survival data shown in C, E and G were analyzed with Mantel-Cox log-rank test. *p*<0.05 was considered significant (* *p*<0.05, ** *p*<0.01, *** *p*<0.001). n = 10 mice per group.

To analyse the relevance of inflammatory monocytes *versus* neutrophils in *P*. *berghei ANKA* induced ECM in C57BL/6 mice, we used mAb to either deplete (i) Ly6C^+^ and Ly6G^+^ cells by anti-Gr-1 mAb as a comparison to previous studies [21], or (ii) to selectively deplete either Ly6G^+^ neutrophils (anti-Ly6G mAb) [19], or (iii) Ly6C^hi^ inflammatory monocytes with the help of anti-CCR2 mAb [20]. Groups of PbTg-infected mice received the respective depletion antibodies either directly upon PbTg infection (d0) or during infection (days 3 and 5 p.i.). The depletion efficacy of inflammatory monocytes (see Fig 1C for gating, box M, upper left, defined by CD11b^+^Ly6C^hi^Ly6G^-^) or neutrophils (see Fig 1C, box N, upper right, defined by CD11b^+^Ly6C^int^ Ly6G^+^) was confirmed in the blood of all groups in comparison to naïve animals and control-infected mice 24h after mAb application (Fig 2A). These injection regimens led to reduction in CD11b^+^ leukocytes (Fig 2A upper row). We observed an effective elimination of both desired populations in the blood of anti-Gr1-administered mice (Fig 2A, lower row, middle plot). Administration of anti-Ly6G mAb, however, resulted in the depletion of neutrophils, but not of inflammatory monocytes (Fig 2A, lower row, right). In contrast, administration of anti-CCR2 mAb resulted in a strong reduction of Ly6C^hi^CD11b^+^ inflammatory monocytes, but not of Ly6G^+^ neutrophils (Fig 2A, lower row far right).

Next, we proceeded to monitor the effects of depletion on ECM development. Depletion of Ly6C^hi^ monocytes and Ly6G^+^ neutrophils by the three different mAbs lasted for at least 48h in all groups (S2 Fig). Therefore, we injected the appropriate mAbs either on the same day of PbTg infection (day 0) or on days 3 and 5 p.i. in order to evaluate the relevance of the different mononuclear cell populations at both the beginning of infection and during an ongoing infection but before the onset of ECM.

Since the onset of ECM in the non-treated PbTg-infected C57BL/6 mice commonly occurs on day 6 p.i., we depicted the ECM score of all untreated and treated groups of PbTg-infected mice on that day (Fig 2B, 2D and 2F). As expected, control-infected C57BL/6 mice, which did not receive any depleting antibodies, presented strong ECM specific symptoms on day 6 p.i. (Fig 2B, 2D and 2F, black bars), and showed rapid disease progression (Fig 2C, 2E and 2G, closed boxes). Depletion of both inflammatory monocytes and neutrophils by mAb anti-Gr1 on the day of infection significantly prevented the severity of ECM (Fig 2B *c*.*f* bars 1 and 2) and led to continued survival of the infected mice (Fig 2C *c*.*f* square symbols with open circles). Similar significant results regarding the ECM score on day 6 p.i. were observed when anti-Gr1 mAb was injected during ongoing infection (d3+5) (Fig 2B). However, this “late” application did not protect those mice, since we observed only a delay in ECM pathology (Fig 2C). Importantly, selective depletion of neutrophils through administration of anti-Ly6G mAb only marginally influenced the developing pathology (Fig 2D *c*.*f* bars 1 and 2) and survival (Fig 2E) and this was regardless of the mAb administration time point. In agreement with anti-Gr1 injection, only the early depletion of Ly6C^+^ inflammatory monocytes through the administration of anti-CCR2 mAb resulted in a significantly reduced ECM score at day 6 p.i. (Fig 2F *c*.*f*. bars 1 and 2), as well as an enhanced survival (Fig 2G). In contrast, injection of anti-CCR2 mAb during an already ongoing infection barely influenced developing pathology and did not protect against ECM-related death (Fig 2E *c*.*f*. bars 1 and 3). Importantly, parasitemia levels did not differ significantly between the experimental groups with and without depletion approaches (S1 Fig). In conclusion, even if anti-Gr1 mAb-mediated depletion of both inflammatory monocytes and neutrophils resulted in the best protection of PbTg-infected mice from ECM, the selective depletion of inflammatory monocytes, but not neutrophils was sufficient to achieve significant protection against ECM.

### Administration of anti-CCR2 or anti-Gr1 mAbs prevents lymphocyte infiltration into the brains of PbTg infected mice

**Fig 3:**
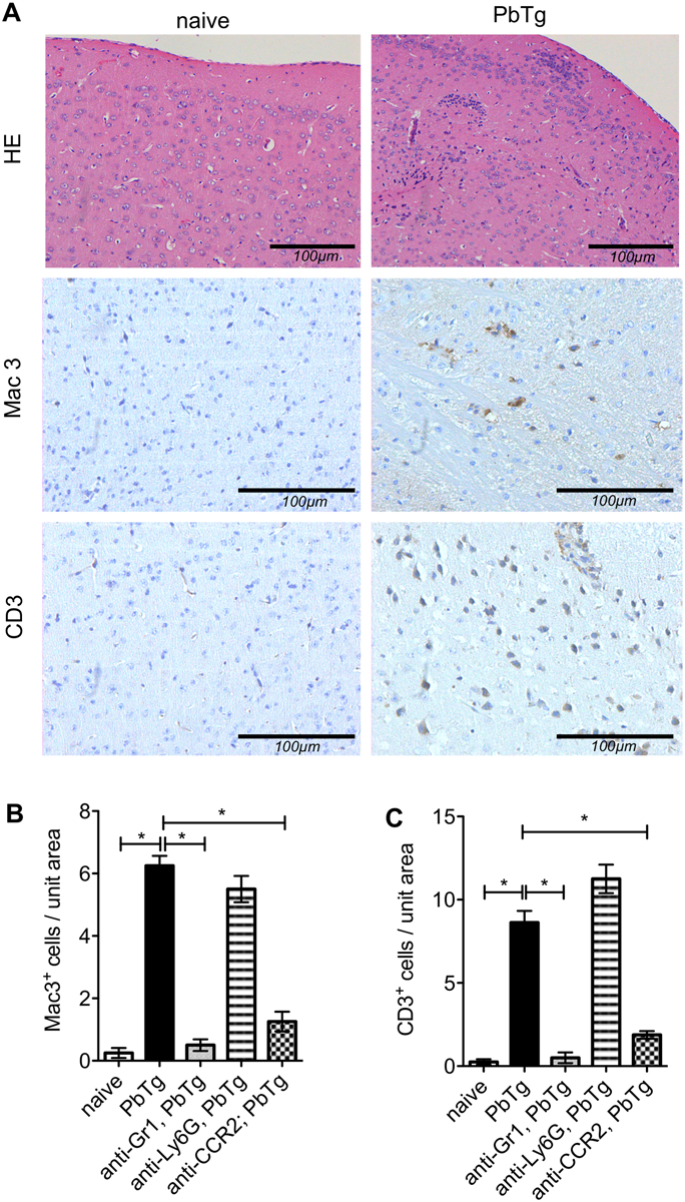
Histological analysis of brains of PbTg infected mice upon early depletion. C57BL/6 mice were infected i.v. with 5*10^4^ PbTg-iRBC and then subdivided into groups that received either anti-Gr1, anti-Ly6G or anti-CCR2 mAb on d0 p.i. (early depletion). On day 6 p.i., tissue sections from the brain parenchyma of individual mice were assessed for pathological changes. (A) Standard H&E staining is depicted in the upper panels whereas immunohistochemical staining for anti-Mac3 and anti-CD3 are shown in the middle and lower panels, respectively. Representative sections from naïve mice are shown on the left whereas those from PbTg infected mice are displayed on the right. (B, C) Quantification of Mac3^+^ cells (B) and CD3^+^ cells T cells (C) in brain sections from the meninges and frontal cortex of individual mice. Scale bars indicate 100μm in the magnifications. Bars show mean ± SEM from n = 8 mice per group from 1 out of 3 independent depletion-infection experiments counted in 10 defined fields (High power fields, HPF) in the frontal cortex. Statistical analysis was performed using Kruskal-Wallis test and Dunn’s Post test and significant differences are indicated by the stars in brackets between the groups (* *p*<0.05).

Next, we determined whether the selective depletion of these immune cell subsets using mAb also altered the amount of lymphocyte infiltration into the CNS since this has also been shown to play a decisive role in the outcome of murine malaria [6, 8]. At the peak of ECM incidence in PbTg infected C57BL/6 mice (day 6 p.i.), tissue sections from the brains of naïve and infected mice were analysed for the number of infiltrating mononuclear phagocytes (Mac3 staining) and T cells (CD3 staining). Fig 3A shows representative stainings from brain frontal cortex prepared from naïve and PbTg-infected mice, respectively. As expected, we detected a strong increase in infiltrating cells into the CNS parenchyma of infected mice compared to naïve controls (representative images in Fig 3A, upper right). These CNS infiltrates consisted of increased numbers of CD11b^+^ mononuclear cells and CD3^+^ T cells (Fig 3A, 3B and 3C). Early depletion of inflammatory monocytes in the blood by using anti-Gr1 or anti-CCR2 mAb resulted in significantly reduced the numbers of both infiltrating mononuclear cells and T cells in the frontal cortex (Fig 3B and 3C *c*.*f*. bars 2 with 3 and 5). No differences in the number of infiltrating lymphocytes were observed when infected mice were subjected to treatment with anti-Ly6G mAb (Fig 3B and 3C *c*.*f*. bars 2 with 4). Depletion using anti-Gr1 and anti-CCR2 but not anti-Ly6G during ongoing PbTg infection on days 3 and 5 also reduced the influx of immune cells as shown by immunohistochemistry, but to a lesser extent (S2 Fig). Therefore, these data showing a massive reduction in lymphocyte influx into the brains of anti-Gr1 or anti-CCR2 mAb-injected mice strongly support our observations described above, that using anti-Gr1 or anti-CCR2 mAb at the time point of infection prolonged the survival of *Plasmodium* infected mice (Fig 2C and 2G).

**Fig 4:**
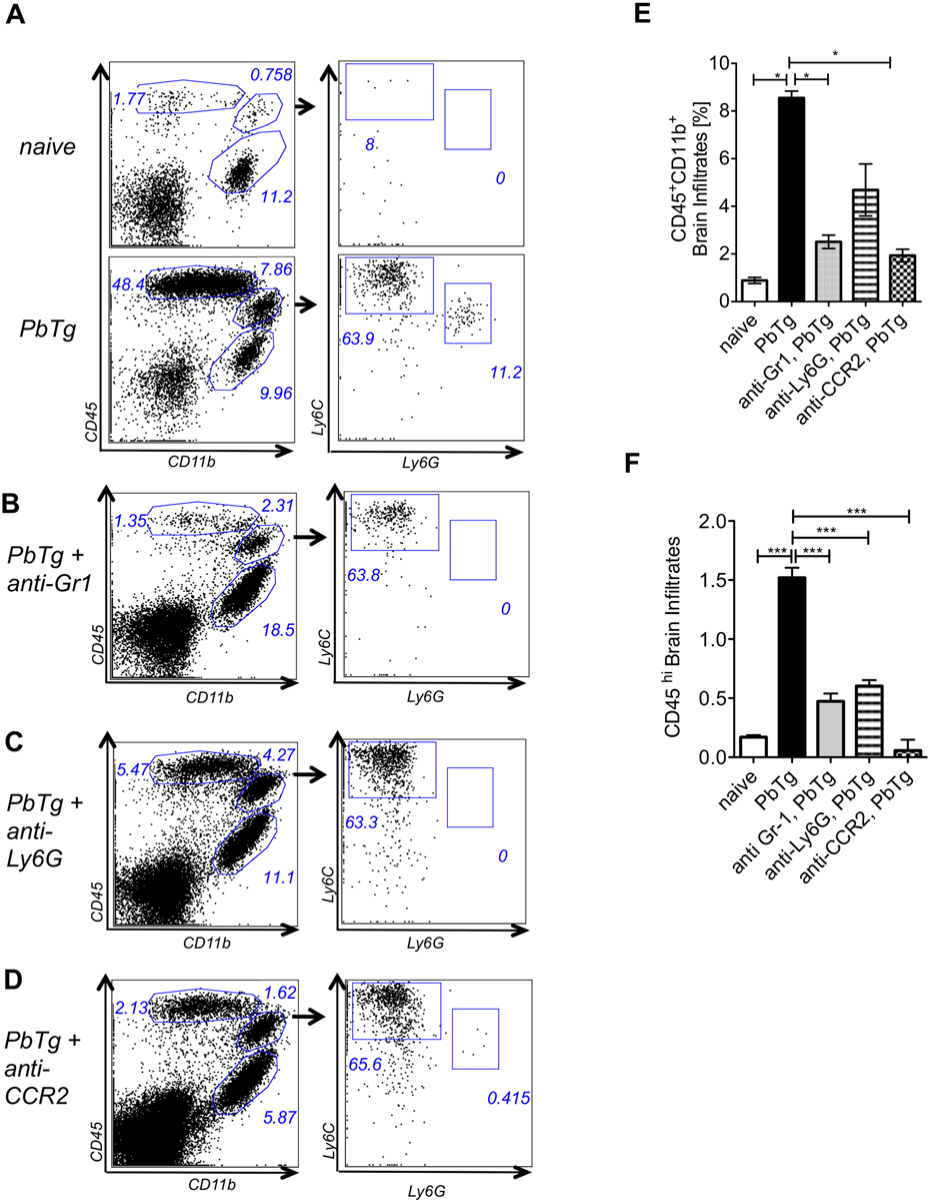
Early monocyte depletion prevents lymphocyte infiltration into the brain. C57BL/6 mice were left either untreated or infected with 5*10^4^ PbTg iRBC (A). In addition, groups of infected mice were treated either with anti-Gr1 (B), anti-Ly6G (C) or anti-CCR2 (D) mAb on the day of PbTg-infection (day 0). Six days later, cellular infiltrates from the brains of individual mice were prepared and analysed for the frequency of (E) infiltrating mononuclear cells (CD45^+^CD11b^+)^ as well as (F) CD45^hi^ lymphocytes (CD45^hi^CD11b^-^). Bars show mean ± SEM from n = 4–5 mice per group from 1 out of 3 independent depletion-infection experiments. Statistical analysis was performed using Kruskal-Wallis test and Dunn’s Post test and significant differences are indicated by the stars in brackets between the groups (* *p*<0.05).

To evaluate how the depletion of inflammatory cell subpopulations in the periphery could influence cellular infiltration into the CNS in more detail, we further determined on day 6 p.i. the frequency and composition of infiltrating cells within the brains of PbTg-infected mice by flow cytometry. When compared to naïve mice, PbTg infected animals exhibited in the brains strongly elevated frequencies of CD45^+^CD11b^-^lymphocytes as well as CD45^+^CD11b^+^ mononuclear cells (Fig 4A, upper and lower panel). Within the CD11b^+^ mononuclear cells in brains of PbTg-infected mice, we found Ly6C^+^Ly6G^-^ inflammatory monocytes and Ly6C^int^Ly6G^+^ neutrophils (Fig 4A, compare upper and lower right plots). Upon anti-Gr1 mAb treatment, we detected a decrease in infiltrating cells which included 3 fold less infiltrating CD45^+^CD11b^+^ cells and a massive reduction in infiltrating CD11b^-^CD45^hi^ lymphocytes compared to WT infected control mice (Fig 4B), which correlates to our findings in peripheral depletion (Fig 2A) and the observed protection (Fig 2B and 2C) and previous studies [21]. Interestingly, the frequency of inflammatory monocytes within the parental mononuclear CD45^+^CD11b^+^ subset was comparable to control infected animals (Fig 4A and 4B; 63.8% vs 63.9.4%), but compared to WT infected control mice, the overall frequency of mononuclear cells was strongly reduced in brains of mAb treated mice (Fig 4E and 4F). These population changes were even stronger when infected mice were injected with anti-Gr1 mAb on days 3 and 5, demonstrating an enduring depletion effect upon administration of the mAb (S3 Fig).

Application of the neutrophil depleting mAb anti-Ly6G also resulted in the reduction of infiltrating mononuclear CD11b^+^ cells as well as CD45^hi^ lymphocytes, which differs to our finding with the help of immunohistochemistry from the brain frontal cortex (Fig 3C), although the overall infiltration of CD45^hi^ cells upon anti-Ly6G injection occurred to a lesser degree compared to the other depletion approaches (Fig 4C and 4E, S3 Fig). Finally, cell preparations from the brains of mice that received anti-CCR2 mAb also contained lower amounts of infiltrating CD45^+^ cells. Here the proportion of CD45^hi^ lymphocytes was also strongly reduced (Fig 4D and 4F). Animals that received applications of the anti-CCR2 antibody on days 3 and 5 after infection, showed decreased amounts of lymphocytes and especially Ly6C^hi^ monocytes (S3 Fig). However, infiltrating neutrophils were barely detected in the brains and this was regardless of the therapy protocol.

Thus, either depletion approaches, anti-Gr1 or anti-CCR2 mAb, if performed early in infection, inhibited the development of ECM, which is paralleled by decreased infiltration of peripheral immune cells into the brains of PbTg infected mice. Even if the effects of late injections of the individual depleting antibodies on days 3 and 5 could be noticed in the brain, and also anti-Ly6G mAb-injected mice showed less infiltrates as shown here by flow cytometry, nearly no one of these mice were protected against ECM. All late depletions and anti-Ly6G depletion approaches that were analysed here showed a delay of the disease onset but no protection from ECM. Our data from early anti-CCR2 mAb administration show that the selective depletion of Ly6C^hi^ monocytes induced effects in the brain that were comparable to protective anti-Gr1 mAb administration, indicating an important role of Ly6C^hi^ monocytes but not neutrophils in ECM development during PbTg infection.

### Depletion of Ly6C^hi^ inflammatory monocytes results in reduced infiltration of IFNγ producing cells into the CNS

**Fig 5:**
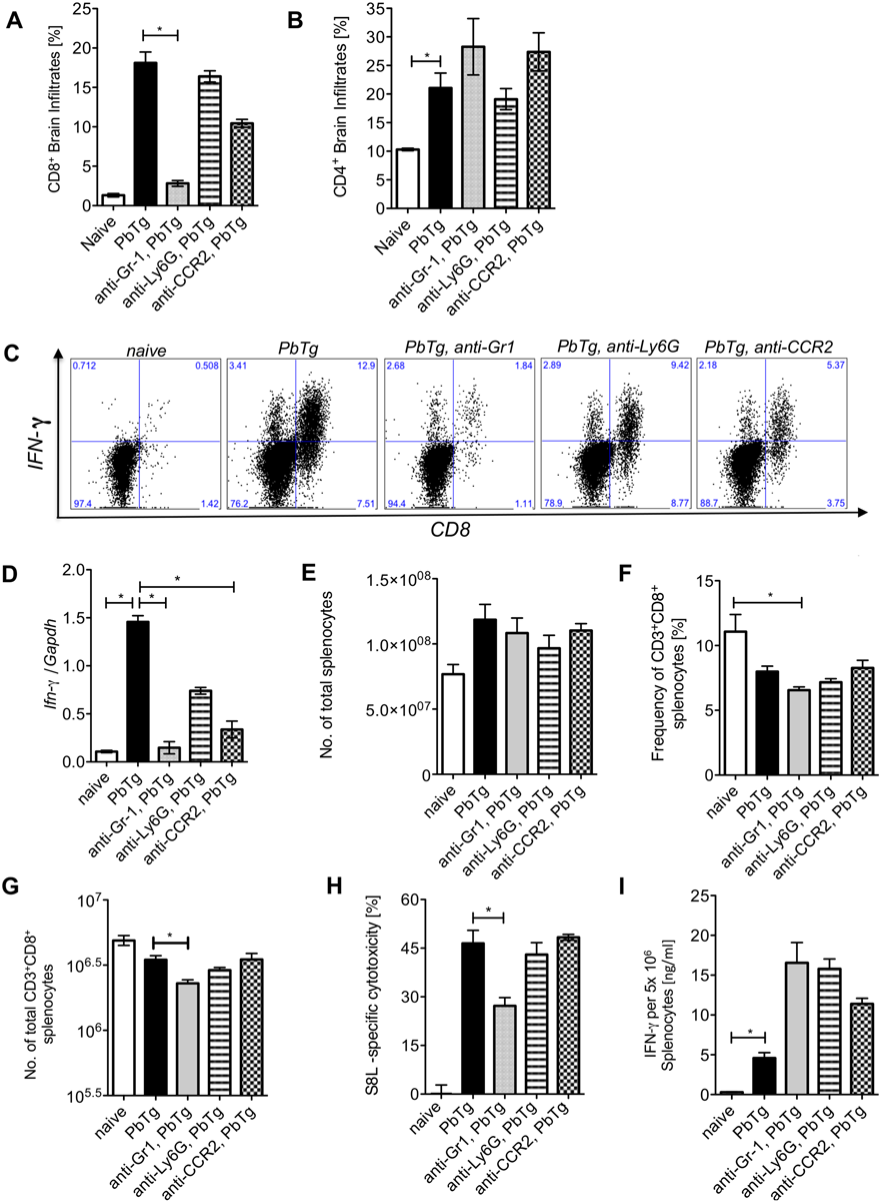
Early monocyte depletion prevents IFNγ producing T cell infiltration into the brain but does not affect peripheral PbTg-specific CTL responses. C57BL/6 mice were infected with 5*10e4 PbTg iRBC i.v.. Simultaneously, groups of mice received either anti-Gr1, anti-Ly6G or anti-CCR2 mAb. Six days later, T cell subsets in the brain and spleen were analysed and cellular immune responses were evaluated. (A) Frequencies of CD8^+^ cells and (B) CD4^+^ cells among brain infiltrates were determined by flow cytometry. Each group contained 4–5 mice. (C) Leukocyte preparations from the brains of individual mice were re-stimulated *ex vivo* and intracellular stained to determine the frequency of IFNγ producing CD8^+^ T cells. Representative images of brain derived IFNγ^+^CD8^+^ T cells by flow cytometry are shown. Calculated frequencies of CD8^+^ IFNγ^+^ T cells and CD8^+^ IFNγ^+^ T cells in the brains as analysed from (A) are shown in Table 1. (D) Fold increase of IFNγ mRNA levels relative to GAPDH in the brains of PbTg-infected mice ± d0 depletion on day 6 p.i. n = 6–8 per group, Kruskal Wallis test with Dunn’s Post test was performed. (E) Total cell count of splenocytes from all d0 depletion groups and controls at day 6 p.i. (F) Frequency of CD3^+^CD8^+^ splenocytes in percent from all d0 mAb depletion groups and controls at day 6 p.i. (G) Calculated total amount of CD8^+^ splenocytes according to data from F and G. (H) *In vivo* cytotoxicity assay analysing PbTg-specific T cells. Recipient PbTg-infected mice, that were or were not additionally treated with depletion antibodies, received an adoptive transfer of CSFE-labelled T cells, that were loaded with ovalbumin-derived MHC class I peptide SIINFEKL and unloaded control cells i.v. (1x10^7^/mouse) on day 5 p.i.. 18 hours later the level of lytic activity was measured by flow cytometry in the spleen. (I) In addition, splenocytes from the same animals as in (H) were re-stimulated with SIINFEKL *ex vivo* for 24 hours and IFNγ production was quantified by sandwich ELISA. (A-I) Bars show mean ± SEM from n = 4–5 mice per group from 1 out of 3 independent depletion-infection experiments. Statistical analysis was performed using Kruskal-Wallis test and Dunn’s Post test and significant differences are indicated by the stars in brackets between the groups (* p<0.05, ** p<0.01).

To determine whether the reduced number of CD3^+^ T cells, which we observed in immunohistochemistry in the frontal cortex of anti-Gr1 or CCR2 mAb PbTg-infected mice also affected different T cell populations that cross the blood brain barrier, we analysed the frequencies of CD8^+^ and CD4^+^ T cells in the preparations from the whole brains by flow cytometry. In addition, we analysed the capacity of CD8^+^ T cells to produce IFNγ. On day 6 p.i., PbTg infected mice presented elevated frequencies of CD8^+^ T cells and CD4^+^ T cells in the brain when compared to naïve mice (Fig 5A and 5B *c*.*f*. bars 1 and 2). More than half of the CD8^+^ T cells that had infiltrated the brains of PbTg-infected mice, produced IFNγ (Table 1, Fig 5C). Administration of anti-Gr1 at the start of PbTg infection resulted in a significant reduction of CD8^+^ T cells, including IFNγ producing and non-producing CD8^+^ T cells (Table 1) but did not change the influx of CD4^+^ T cells within the brain (Fig 5A and 5B *c*.*f*. bars 2 with 3). These results were also observed when we administered antibodies on days 3 and 5 of infection (Table 1). Importantly, administration of anti-CCR2 mAb led to significantly reduced frequencies of IFNγ producing CD8^+^ T cells in the brains of the mice, whereas the frequencies of CD4^+^ T cells were not changed (Table 1). The depletion of neutrophils however, using anti-Ly6G mAb, did neither result in reduced frequencies of CD8^+^ T or CD4^+^ T cells nor in the changed proportion of IFNγ producing CD8^+^ T cells in the brain compared to non-depleted infected mice (Fig 5A and 5B *c*.*f*. bars 2 and 4, Fig 5C, Table 1). Thus, we conclude that the protection of PbTg-infected mice from ECM upon depletion of Ly6C^hi^ monocytes by anti-Gr-1 or anti-CCR2 mAb was due to abrogated infiltration of lymphocytes into the CNS, mainly of IFN-γ-producing CD8^+^ T cells. Next, we analysed the expression of IFNγ in the brains of PbTg-infected and depleted mice. We detected elevated mRNA levels of IFNγ in the brains of PbTg-infected mice (Fig 5D *c*.*f*. bars 1 and 2), which were 10-fold reduced in the brains of anti-Gr1 mAb treated infected mice and five-fold reduced in the brains of anti-CCR2 treated infected mice (Fig 5D *c*.*f*. bars 2 with 3 and 5). Administration of anti-CCR2 or anti-Gr-1 mAb during an ongoing PbTg infection also resulted in reduced IFNγ levels in the brains, which was significant in the case of anti-Gr-1, demonstrating again a strong effect of this clone (S4 Fig). Interestingly, the d0 application of anti-Ly6G mAb to infected mice resulted in a two-fold decrease of IFNγ levels in the brain, but remained a trend (Fig 5D *c*.*f*. bars 2 and 4), similar to day 3 and 5 application of anti-Ly6G (S4 Fig 4). Taken together, we observed the strongest effects concerning influx of T cells into the brain and block of IFNγ production in anti-Gr-1 mAb injected mice, but importantly, comparable effects were achieved with the selective depletion of Ly6C^hi^ inflammatory monocytes with the help of anti-CCR2-injection.

**Table 1:** Impact of mononuclear cell subset depletion on IFNγ producing CD8^+^ T cells (brain infiltrates).

	CD8 positive Infiltrates (Brain)	
Experimental group ^a^	IFNγ positive [%]	IFNγ negative [%]
Naive	0,494 ± 0,263	0,82 ± 0,328
PbTg-infected	10,360 ± 1,744 ^b^	7,76 ± 1,155 ^b^
PbTg, anti-Gr1, d0	1,474 ± 0,323 ^c^	1,35 ± 0,402 ^c^
PbTg, anti-Ly6G, d0	8,293 ± 1,621	7,02 ± 1,419
PbTg, anti-CCR2, d0	5,258 + 1,069 ^c^	3,78 ± 1,190 ^c^
PbTg, anti-Gr1 d3+5	1,463 ± 0,784 ^c^	1,14 ± 0,605 ^c^
PbTg, anti Ly6G d3+5	8,793 ± 2,610	6,02 ± 1,383
PbTg, anti-CCR2 d3+5	8,923 ± 1,939	5,14 ± 0,959

Six days after infection, cellular infiltrates from the brains of individual mice were prepared and analysed by flow cytometry for the frequency of infiltrating CD8^+^ cells which produce IFNγ or not upon phorbol myristate acetate (PMA) /Ionomycin restimulation.

^a^ n = 4–5 per group

^b^ Significant differences between PbTg infected group and naïve mice, (p<0.05, Mann-Whitney U test)

^c^ Significant differences between mAb-depleted versus non-depleted PbTg infected mice, (p<0.05, Mann-Whitney U test)

### Specific depletion of Ly6C^hi^ inflammatory monocytes and neutrophils did not prevent the generation of antigen-specific CTL responses

We observed that PbTg-infected mice, which were depleted of inflammatory monocytes, lacked lymphocyte infiltration into the brain, and from this finding we hypothesized that early depletion interfered with the generation of functional T cell responses in the periphery. We determined first whether the cell subset depletion by the different mAbs had an impact on the total cell number in the spleen or on T cell frequencies and numbers during the early phase of infection and analysed all experimental groups on day 2 after PbTg infection with and without mAb depletion. We determined on day 2 after mAb-injection and PbTg infection in blood and spleens of all PbTg-infected mice that received any depleting antibody, a reduction in CD11b^+^ leukocytes (S5 Fig). In spleens of mice that received anti-Ly6G mAb or anti-CCR2 mAb, significantly less Ly6G^+^ neutrophils and Ly6C^+^ monocytes (S5 Fig), respectively, were present among those CD11b^+^ cells. In contrast, anti-Gr1 mAb clone RB6 did not only result in depletion of both Ly6C^+^ monocytes and Ly6G^+^ neutrophils, but also in significant reduction of CD3^+^CD8^+^ T cells, as observed on day 2 after infection and mAb injection (S5 Fig).

We analysed then the spleens of all experimental groups on day +6 after infection and observed, that the cell counts of splenocytes from all d0 depleted groups of mice were comparable to naïve and control-infected littermates (Fig 5E). In contrast, total splenocytes counts from d3+5 anti-Gr1 mAb depleted mice were significantly reduced (S4 Fig). Frequencies and total numbers of CD8^+^ T cells were reduced in all infected mice compared to naïve mice and reached significance in the anti-Gr1 injected group of mice (Fig 5F and 5G) as well as in the late depletion groups of anti-Gr1 mAb (S4 Fig). As this might have an impact also on parasite control mechanisms in the periphery, we performed an *in vivo* cytotoxicity assay using an adoptive transfer of fluorescent-labelled and OVA-peptide loaded spleen cells to evaluate the antigen-specific cytotoxic capacity of CD8^+^ T cells. Remarkably, functional CTL responses were present in all groups, although infected mice treated with anti-Gr1 mAb on the day of infection presented a reduced lytic ability of Ag-specific CTLs (Fig 5H *c*.*f*. bars 2 and 3), whereas the CTL responses of d3+5 depleted mice did not differ significantly from the control-infected group of mice (S4 Fig). Interestingly, we did not determine any deficits regarding the capacity of the T cells to release IFNγ upon peptide-specific restimulation between the different depleted groups and the control-infected groups of mice, neither upon mAb administration at the time point of infection nor during ongoing infection (Fig 5I, S4 Fig). Here, we concluded that all PbTg-infected groups were equally able to generate functional antigen-specific T cell responses except anti-Gr1 treated mice, as here the IFNγ responses have to be set off against the strongly reduced CD8^+^ T cell numbers. Thus, the depletion of inflammatory monocytes or neutrophils with the help of selectively recognizing mAb did not prevent the generation of antigen-specific CTL responses in PbTg-infected mice, whereas application of anti-Gr1 significantly impaired CTL responses in the periphery.

## Discussion

Cells of the innate immune system are essential for the detection and successful elimination of invading pathogens. They are also important for the induction of powerful adaptive immune responses, which aim at the complete clearance of pathogenic microbes. However, strong inflammatory immune responses harbour the risk of harming the host and therefore should be prevented or regulated. Infection with *Plasmodium* parasite are a good example of the possibility of overwhelming inflammatory immune responses, since *P*. *falciparum* infections in infants and non-immune travellers are often fatal because they lead to cerebral malaria and other complications, whereas re-infected adults from endemic areas show successfully dampened immune responses and a mild course of disease. ECM, the pathological outcome of experimental infection with *P*. *berghei ANKA* in C57BL/6 mice, is a result of IFNγ production and CD8^+^ T cell infiltration into the CNS [6, 8]. Here, we provide initial evidence that Ly6C^hi^ inflammatory monocytes are essential immune players in PbTg infection since specific depletion by injection of anti-CCR2 mAb protected PbTg-infected mice from ECM pathology. Moreover, depletion of this cell subset reduced both cellular infiltration to and IFNγ levels in the CNS. Importantly, this selective elimination of Ly6C^hi^ monocytes did not prevent the generation of *Plasmodium*-specific immune responses in the periphery, as shown by effective CTL responses and IFNγ production, which might be essential for parasite elimination.

The indispensable requirement of innate immune cells i.e. monocytes, macrophages and neutrophils, in a T-cell-driven disease such as malaria became apparent after the general depletion of phagocytic cells using CloLip, which resulted in prolonged survival of PbTg-infected C57BL/6 mice (Fig 1). Our analysis here revealed that upon clodronate liposome-mediated depletion mainly F4/80 positive cells were absent, which were also positive for Ly6C, strongly suggesting that these cells were inflammatory monocytes. However, data from previous studies highlighted a role for F4/80^-^Ly6G^+^ neutrophils in ECM [21].

Until recently, an exclusive *in vivo* depletion of these two immune cell populations, Ly6C^+^ monocytes and Ly6G^+^ neutrophils, was not achievable. However, we previously reported the necessity to properly distinguish between monocytes and neutrophils in *T*. *gondii* infections using the anti-Ly6G clone 1A8 [9, 11, 12, 24]. Indeed, those observations revealed the critical importance of Ly6C^hi^ inflammatory monocytes during an acute infection with *T*. *gondii* and clearly demonstrated that neutrophils are not protective as reported previously, but rather contribute to the pathology [9, 11].

According to current studies, it is acknowledged that anti-Gr1 recognizes different Ly6 isoforms, in particular Ly6G as well as Ly6C, resulting in a broad depletion spectrum, including Ly6C monocytes, but also certain lymphocyte subsets. Therefore, the question arose about how valid the commonly used anti-Gr1 clone RB6 was in selectively depleting neutrophils, as claimed in several previous reports. For this reason, in this study we utilized anti-Ly6G and anti-CCR2 mAbs to selectively deplete either neutrophils or inflammatory monocytes and compared those to mice that received anti-Gr1 mAb. To investigate whether and at which time point therapeutic intervention regarding ECM onset was possible, PbTg infected mice received the depleting mAb either on the day of infection or during infection. We show clearly that Gr-1 mAb depletes both Ly6C^hi^ monocytes and Ly6G^+^ neutrophils, but however, injection of anti-Gr1 did result also in significant reduction of CD3 T cells, which was observed during the early phase of infection, but lasted until day 6 after infection. From these data and the observations in regard to ECM development we conclude that the protective effect of anti-Gr1 mAb-mediated depletion was probably mediated by the early depletion of inflammatory monocytes but not of neutrophils and most likely also by the depletion of T cells. We come to this conclusion since anti-CCR2 injection, but not anti-Ly6G injection resulted in significant protection of PbTg infected mice. In addition, the protective effect of T cell depletion is well described in the literature [6]. This protection from ECM was characterized by dampened tissue-specific brain inflammation, but importantly, an anti-CCR2 mAb injection did not prevent peripheral immune responses. In contrast, anti-Gr1 mAb injection diminished the frequency and function of peripheral T cells.

We additionally provide immunohistochemical evidence showing that the number of infiltrating CD3^+^ T lymphocytes within the brain during infection was significantly reduced in mice where inflammatory monocytes were absent. Moreover, on day 6 *p*.*i*., T cells and also functional immune responses could be detected in the spleens among all groups of mice which had received depleting antibodies either on day 0 of infection or days 3 and 5. Consequently, we hypothesize that Ly6C^hi^CCR2^pos^ inflammatory monocytes are crucially involved in the early phase of *Plasmodium* blood-stage infection. It remains to be analysed, at which step of infection and how the monocytes interact with other immune cells to trigger T cell influx into the brain.

Importantly, an essential role for Ly6C^hi^ monocytes in the control of blood-stage malaria parasites was shown in the non-ECM murine model for malarial anaemia using *P*. *chabaudi* [25]. Sponaas et al. demonstrated CCR2-dependent egress of monocytes from the bone marrow and in infected CCR2-deficient mice a strong reduction of parasitemia in the acute stage of infection due to an adoptive transfer of splenic Ly6C^hi^ monocytes. The authors further concluded that such control over the blood stage of infection was mediated through the production of reactive oxygen intermediates and inducible nitric oxide synthase by these monocytes [25]. Our data did not reveal significant differences in parasitemia levels between the experimental groups; although anti-Gr1 mAb injected mice presented slightly increased parasitemia levels compared to the control-infected mice, this remained a trend and did not reach significance.

In addition, our data suggest, that Ly6C^+^CCR2^+^ inflammatory monocytes do not prime parasite-specific T cells, as the anti-CCR2 injected mice generated T cell responses in the periphery that were comparable to those of control-infected mice. In contrast, anti-Gr1 mAb injection did result in strongly diminished T cell frequencies and also numbers, which was also observed before. We show here for the first time, that anti-Gr1 treatment also reduced CTL responses. Several recent studies have reported that the administration of this mAb results in the non-selective depletion of monocytes [19, 26, 27]. For example, Daley et al. showed loss of monocyte/macrophages upon anti-Gr1 mAb clone RB6 application in a wound model and endotoxemia [19]. Wojtasiak et al. reported the depletion of several immune cells using this anti-Gr1 mAb in a herpes simplex virus type 1 infection [26]. In addition, Johnson et al. used the peptide-induced fatal syndrome (PIFS) model of CNS vascular permeability, which depends on CD8^+^ T cell-mediated break down of the blood-brain barrier. In their study, the depletion efficacy of the anti-Gr1 mAb RB6 clone was compared to the mAb 1A8 variant, which is anti-Ly6G. There, Johnson et al. observed a strong reduction of CD45^+^Gr1^+^Ly6G^+^ cells, but also diminished infiltration of Gr1^+^CD8^+^ T cells upon administration of anti-Gr1 clone RB6-8C5, whereas application of the anti-Ly6G mAb resulted in selective neutrophil depletion and did not affect T cell infiltration. They concluded that application of this anti-Gr1 mAb directly depleted infiltrating T cells and that BBB disruption in their model was independent of neutrophils [27]. However, they did not analyse the impact of depletion on migrating T cell populations or antigen-specific T cell responses in the periphery.

Monocytes require CCR2 to leave the bone marrow and therefore—to substantiate our findings—we compared the outcome of PbTg infection using anti-CCR2 mAb as well as anti-Gr1. This antibody has been used to specifically target CCR2^+^ monocyte subsets in models of toxoplasmosis [28], arthritis [29, 30] and gram negative bacterial meningitis [31]. The role of CCR2 in ECM has been analysed in previous studies which focused on infiltrating T cells [32]. Belnoue and colleagues showed that PbA-infected CCR2 KO mice still developed ECM and no differences in infiltrating T cell numbers were observed in the brain. They did not observe any difference in brain infiltrating T cell numbers but instead found a reduced amount of macrophages amongst brain-sequestered leukocytes, compared to infected WT. Contrary to our observation that 50% of mice were protected against ECM by CloLip-mediated macrophage depletion at day 4 p.i. (data not shown), in their study, only depletion of CD8^+^ T cells but not macrophages (at day 5 p.i.) did result in infected mice in protection against ECM [32]. In that study, Belnoue et al. showed an impact of CCR5 on T cell infiltration upon PbA infection, however, a crucial analysis of monocyte subpopulations or functional T cell responses (i.e. production of IFNγ or antigen-specific lysis), which we have done here, was not performed. The disparate observations between our results and what has been previously described by Belnoue et al. may be explained by the possible development of a compensatory mechanism in the genetically modified mice lacking CCR2 that would not be present in mAb immune cell depleted mice in our study. Importantly, we demonstrate that in our study, anti-CCR2 treatment resulted in diminished recruitment of CD8^+^ T cells into the brain, but not in overall T cell depletion as we detected unaffected T cell frequencies 2 days after the depletion and also a robust T cell response in the spleen of CCR2-depleted mice at day 6 p.i., which was determined by CTL activity and IFNγ production upon peptide-restimulation. We assume that PbA—infected CCR2-deficient mice have a different T cell pool that is recruited to the brain due to compensatory mechanisms, which may show a different migration pattern than in infected WT littermates.

In conclusion, our data show that the selective depletion of Ly6C^hi^CCR2^+^ monocytes is able to prevent the development of *P*. *berghei* infection-induced ECM. Their pathogenic phenotype in this model is in agreement with the role these cells have been reported to play in other models including atherosclerosis [33], parasitic infections [34], respiratory fungal infection [35], liver destructive processes [36], obesity [37] and Alzheimer’s disease [38, 39]. Notably, in our study, the selective depletion of these Ly6C^hi^ inflammatory monocytes showed similar protective effects as anti-Gr1 mAb and prevented the crossing of pathogenic immune cells, including monocytes and T cells, into the brains of PbTg infected mice. But in contrast to anti-Gr1 mAb, anti-CCR2 mAb did not disrupt functional antigen-specific T cell responses in the periphery. Moreover, the effects were more enhanced when depletion of Ly6C^hi^CCR2^+^ monocytes occurred early in PbTg infection, which has implications if such strategies were translated to therapeutic platforms. Future studies should determine whether individuals with immunity to malaria have dampened inflammatory monocyte levels in the periphery and their immune-regulatory properties should be analyzed.

## Material and Methods

### Mice

7 week old female C57BL/6N mice were purchased from Janvier (Le Genest Saint Isle, France). Mouse studies were approved by local regulatory agencies (Landesamt fuer Natur, Umwelt und Verbraucherschutz Nordrhein-Westfalen (LANUV NRW) §84–02.04.2012.A264). Water and food were provided *ad libitum*.

### Parasites, infection and disease

In all experiments, transgenic *Plasmodium berghei* ANKA expressing Ovalbumin (PbTg) infected red blood cells (iRBCs) were used to infect mice [22]. All mice were infected intravenously (i.v.) with 5*10^4^ pRBCs obtained from mice that had been previously infected intraperitoneally (i.p.) with stock solution. Stock solution contained 1*10^7^ iRBCs in glycerine from liquid nitrogen storage. Stock mice were of the same background as experimental animals. For evaluation of disease onset mice were monitored twice daily for ECM symptoms and parasitemia. ECM development was scored as described before according to the following symptoms: 0 = without symptoms, 1 = ruffled fur, 2 = hunching, 3 = wobbly gait, 4 = limb paralysis, 5 = convulsions, 6 = coma [40, 41]. Mice reaching score 5 and ECM negative mice, which developed anaemia, were sacrificed due to ethical guidelines. Parasitemia levels in the blood were determined as described [41].

### Reagents and depleting antibodies

For cell culture RPMI 1640 medium (Sigma, Munich, Germany) supplemented with 10% FCS (PAA, Cölbe, Germany), 1% Penicillin/Streptomycin or Gentamicin (Lonza, Wuppertal, Germany), 1% L-Glutamine (PAA, Cölbe, Germany) was used. Directly conjugated monoclonal antibodies used for FACS analysis (anti-mouse CD3, CD4, CD8α, CD11b, CD11c, CD45, CD54, CCR2, I-Ab, F4/80, Ly6C, Ly6G,) were bought from BD Pharmingen (Heidelberg, Germany) and eBioscience (Frankfurt, Germany) and R and D. Phagocytic cells were depleted by i.v. injection of 200μl clodronate liposomes containing 5mg clodronate per ml suspension [23]. Controls received 200μl PBS-filled liposomes i.v. Monocytes and neutrophils together were depleted using the monoclonal antibody (mAb) anti-Gr1 (clone RB6-8C5 from bioXcell, US, NH). Neutrophils were depleted using anti-Ly6G mAb 1A8 [19] (bioXcell, US, NH). Inflammatory monocytes were depleted by the mAb anti-CCR2 (clone MC21) [20]. Monoclonal antibodies were administered by i.p. injection on either day 0 within 30 min of PbA infection (early), or on days 3 and 5 (late) post infection (p.i.). Per mouse, injections comprised of 500 μg for mAb 1A8 (anti Ly6G), 250 μg for mAb RB6 (anti-Gr1) and 75μg for mAb MC21 (anti-CCR2).

### Flow cytometry

For analysis by flow cytometry, mice were anaesthetized and then perfused intra-cardially with 1x PBS. Spleens and brains were isolated, cut into small pieces and digested with 0.5 mg/ml collagenase A (Roche, Basel, Switzerland), by incubation at 37°C for 25 min. Then the organs were gently pressed through a sieve to obtain a single cell suspension and washed with PBS.

To enrich lymphocytes, brain homogenates were resuspended in 5 ml 37% Percoll (GE Healthcare, Freiburg, Germany), which was carefully underlayed with 5 ml of 70% Percoll, respectively. Cells were centrifuged for 20 min at room temperature at 2000 rpm without brake. Cells within the interphase were collected by aspiration through a pipette, transferred into a new tube and washed with 1x PBS / 1% FCS / 2mM EDTA.

Cells were stained in sterile 1x PBS containing 1% FCS for 20 min on ice. For intracellular staining of IFNγ, cells were activated with ionomycin (1 μg/ml), phorbol myristate acetate (50 ng/ml) and Golgi stop (BD Bioscience, Heidelberg, Germany) for 5h. Intracellular stainings were performed with the corresponding staining kits (BD Bioscience, Heidelberg, Germany) according to the manufacturer´s protocol. Acquisition was performed on a FACS Canto II flow cytometer and LSR Fortessa (BD, San Jose, USA). Data were analysed with FlowJo Software (Treestar Inc., Ashland, USA).

### *In vivo* cytotoxicity assay and peptide restimulation

CTL activity in PbTg infected animals was determined *in vivo* on day 6 p.i. as described elsewhere [42]. Briefly, splenocytes from syngenic donor mice were pulsed with 1μM of the specific H-2k^b^ peptide SIINFEKL (Ovalbumin) for 30 min at 37°C and subsequently with 1μM of CFSE for 15 min (CFSE^high^, specific target cells). Reference cells were not pulsed with peptide and labeled with 0.1μM CFSE for 15 min (CFSE^low^, reference cells). After fluorochrome labeling, the cells were washed and the cell number was determined. Then, both cell populations were mixed at a 1:1 ratio (CFSE^high^/ CFSE^low^). Each recipient received 1x10^7^ total cells into the tail vein on day 5. Mice were sacrificed 18 hours later on day 6 after infection and spleens were isolated to prepare single-cell suspensions. Lysis of peptide-loaded cells was quantified by measuring the ratio of CFSE^high^/ CFSE^low^ cells via flow cytometry (Canto II, BD Biosciences). The percentage of specific lysis, termed SL8-specific lysis, was calculated using the following equation: 100 - [(CFSE^high^/CFSE^low^)_immunized_/(CFSE^high^/CFSE^low^)_naïve_] x 100.

### Cytokine ELISA

Spleens from experimental animals were isolated on day 6 p.i. and single cell suspensions were prepared as described above. 10^6^ splenocytes were cultured in triplicate in 200μl RPMI medium including 1μM SIINFEKL peptide over night. The supernatant was analysed for IFNγ by sandwich ELISA according to the manufacture’s guide (eBioscience).

### Histology

Animals were sacrificed on 6 days p.i. and the brains were removed and fixed in 4% neutral buffered formalin. Tissues were dehydrated in ethanol and embedded in paraffin, and 5-μm sections were stained with hematoxylin and eosin (H&E), Mac3 and CD3 as described previously [9, 11]. Quantification of cellular infiltrates in tissue sections of the frontal cortex was performed in 10 high power fields (HPF) as described before [43].

### Real Time PCR

After removal, tissue samples from brains were immediately transferred to RNA later (QIAgen, Hilden, Germany) and kept on ice. They were stored at 4°C for at least 24h and then kept at -20°C until RNA isolation. For RNA isolation, the tissue sample was removed from RNA later and homogenized with 1mL of TriFast (peqGOLD, Erlangen, Germany) in BashingBeads tubes (Zymo Research, Freiburg, Germany). PeqGOLD HP Total RNA Kit was used for purification according to the manufacturer’s instructions. Thereafter, on-membrane DNase I digestion (peqGOLD, Erlangen, Germany) was performed. For qPCR, TaqMan RNA-to-Ct 1-Step Kit (Life Technologies, Darmstadt, Germany) was used with 2 μg total RNA in a reaction volume of 10μL. The following TaqMan Gene Expression Assays (Life Technologies, Darmstadt, Germany) were used to measure mRNA levels for IFNγ: (IFNγ: Mm01168134_m1).

### Statistical analysis

Survival was performed with 10 mice per group and analysed with Log-rank sum test. *Ex vivo* analyses of infection experiments were performed with 4–8 mice per group and statistical analysis was performed non-parametrically using Kruskal-Wallis test with Dunn’s Post test when comparing more than 2 groups; Mann-Whitney U test was used to calculate differences between infected control mice and single antibody-depletion regiments in the analysis of IFNγ production by T cells. If not otherwise stated, mean and standard error of the means are shown. Significant differences are indicated by the stars in brackets between the groups (*p<0.05; **p<0.01, ***p<0.001). All calculations were performed in GraphPad Prism.

## Supporting Information

S1 Fig No difference in parasitemia between infected controls and CloLip-depleted or mAb-depleted mice. Determination of parasitemia levels on day 6 after infection in the blood of mice that had received either (A) Clodronate liposomes or PBS-filled liposomes one day before infection with 5*10^5^ PbTg-iRBCs or (B) mAb-injected mice on day 6 after infection with 5*10^4^ PbTg-iRBCs. The indicated antibodies were injected on the day of infection as described in the main text. N = 6–8 per group, Statistical analysis was performed by student’s *t* test (A) or Kruskal-Wallis test (B).
(TIF)

S2 Fig Late depletion of Ly6C monocytes prevents lymphocyte infiltration into the brain. C57BL/6 mice were infected i.v. with 5*10e4 PbTg-iRBC and then subdivided into groups that received either anti-Gr1, anti-Ly6G or anti-CCR2 mAb on days 3 and day 5 p.i. (late depletion). On day 6 p.i., tissue sections from the brains of individual mice were assessed for pathological changes. Quantification of Mac3^+^ cells (A) and CD3^+^ T cells (B) in brain tissue sections of individual mice. Bars show mean ± SEM from n = 8 mice per group. Statistical analysis was performed using Kruskal-Wallis test and Dunn’s Post test and significant differences are indicated by the stars in brackets between the groups (* p<0.05). HPF, High Power Field.
(TIF)

S3 Fig Monocyte depletion prevents lymphocyte infiltration into the brain. C57BL/6 mice were left either untreated or infected with 5*10e4 PbTg iRBC (see main Fig 4A). (A) In addition, groups of infected mice were treated either with anti-Gr1 (upper plots), anti-Ly6G (middle plots) or anti-CCR2 mAb (lower plots) on day 3 and 5 during PbTg-infection. On day 6 p.i., cellular infiltrates from the brains of individual mice were prepared and analysed for the frequency of infiltrating lymphocytes (CD45^hi^CD11b^-^) and mononuclear cells CD45^+^CD11b^+^ cells and therein the amount of recruited monocytes (Ly6C^+^) and neutrophils (Ly6G^+^) by flow cytometry. Representative plots from one out of four mice are shown. (B) Frequency of CD11b^+^CD45^+^ cells (upper graph) and CD45^hi^CD11b^- cells^ among the brain infiltrates (lower graph). (C, D) CD45^+^CD11b^-^ cells were then assessed for the expression of CD8 and CD4. Bars show mean ± SEM from n = 4–5 mice per group. Statistical analysis was performed using Kruskal-Wallis test and Dunn’s Post test and significant differences are indicated by the stars in brackets between the groups (* p<0.05).
(TIF)

S4 Fig Impact of mononuclear cell subset depletion on cell counts and frequencies of T cells in the spleen. C57BL/6 mice were left either untreated or infected with 5*10e4 PbTg iRBC. In addition, groups of infected mice were treated either with anti-Gr1, anti-Ly6G or anti-CCR2 mAb (on day 3 and 5 during PbTg-infection. (A) Total cell count of splenocytes from all d3+5 depletion groups and controls at day 6 p.i. (B) Frequency of CD8^+^ splenocytes in percent from all d3+5 depletion groups and controls at day 6 p.i. (C) Calculated total amount of CD8^+^ splenocytes according to data from B and C. (D) Fold increase of IFN-γ mRNA levels relative to GAPDH in the brains of PbTg-infected mice ± d3+5 mAb depletion on day 6 p.i. n = 6–8 per group, Kruskal Wallis test with Dunn’s Post test was performed. (E) *In vivo* cytotoxicity assay analysing PbTg-specific T cells at day 6 in the spleens, using SIINFEKL loaded target cells which were adoptively transferred into infected and non-infected mice 18 hours before analysis. (F) Splenocytes from the same animals as in E were re-stimulated with SIINFEKL *ex vivo* for 24 hours and IFN-γ production was quantified by sandwich ELISA.(A-F) Bars show mean ± SEM from n = 4–5 mice per group. Statistical analysis was performed using Kruskal-Wallis test and Dunn’s Post test and significant differences are indicated by the stars in brackets between the groups (* p<0.05).
(TIF)

S5 Fig Analysis of specific depletion in the spleen on day 2 after PbTg infection. C57BL/6 mice were left either untreated or infected with 5*10e4 PbTg iRBC. In addition, groups of infected mice were treated either with anti-Gr1, anti-Ly6G or anti-CCR2 mAb on the day of PbTg-infection. Two days later, mice were sacrificed for analysis. (A) The diagram illustrates on the left panel the gating strategy for leukocytes from spleen and blood used in flow cytometric analysis. The right panel shows further analysis of CD11b^+^ gated splenocytes for expression of Ly6C and Ly6G to identify monocytes and neutrophils, respectively, as well as further analysis of CD3^+^ gated cells for expression of CD4 and CD8. The data show splenocytes from a naïve C57BL/6 mouse. (B) According to the gating scheme shown in (A), splenocytes from all experimental groups were analyzed for the expression of CD3 versus CD11b (left panel). Ly6C^+^ monocytes and Ly6G^+^ neutrophils among the previously gated CD11b^+^ cells are shown in the middle panel, whereas CD3^+^ gated cells were further analyzed for the expression of CD4 and CD8 (right panel). Representative data of each experimental group are shown, one out of four mice. (C) Total splenocytes count (D-O) Flow cytometric analysis of frequencies and calculation of total amounts of splenic subpopulations, which were gated according to the scheme shown in (A). (D) Frequency and (E) total amount of CD11b^+^ splenocytes; (F) Frequency and (G) total amount of Ly6C^+^ monocytes gated from CD11b^+^ splenocytes; (H) Frequency and (I) total amount of Ly6G^+^ neutrophils gated from CD11b^+^ splenocytes; (J) Frequency and (K) total amount of CD3^+^ splenocytes; (L) Frequency and (M) total amount of CD8^+^ T cells gated from CD3^+^ splenocytes, (N) Frequency and (O) total amount of CD4^+^ T cells gated from CD3^+^ splenocytes. N = 4 per group, Statistical analysis was performed by Kruskal-Wallis test with Dunn’s Post test. Significant differences are indicated by the stars in brackets between the groups (** p<0.01).
(TIF)
