# Supplementary figures and images for: Specific Depletion of Ly6Chi Inflammatory Monocytes Prevents Immunopathology in Experimental Cerebral Malaria
Source: PLoS One. 2015 Apr 17;10(4):e0124080. doi: 10.1371/journal.pone.0124080 (PMC4401438; doi:10.1371/journal.pone.0124080)

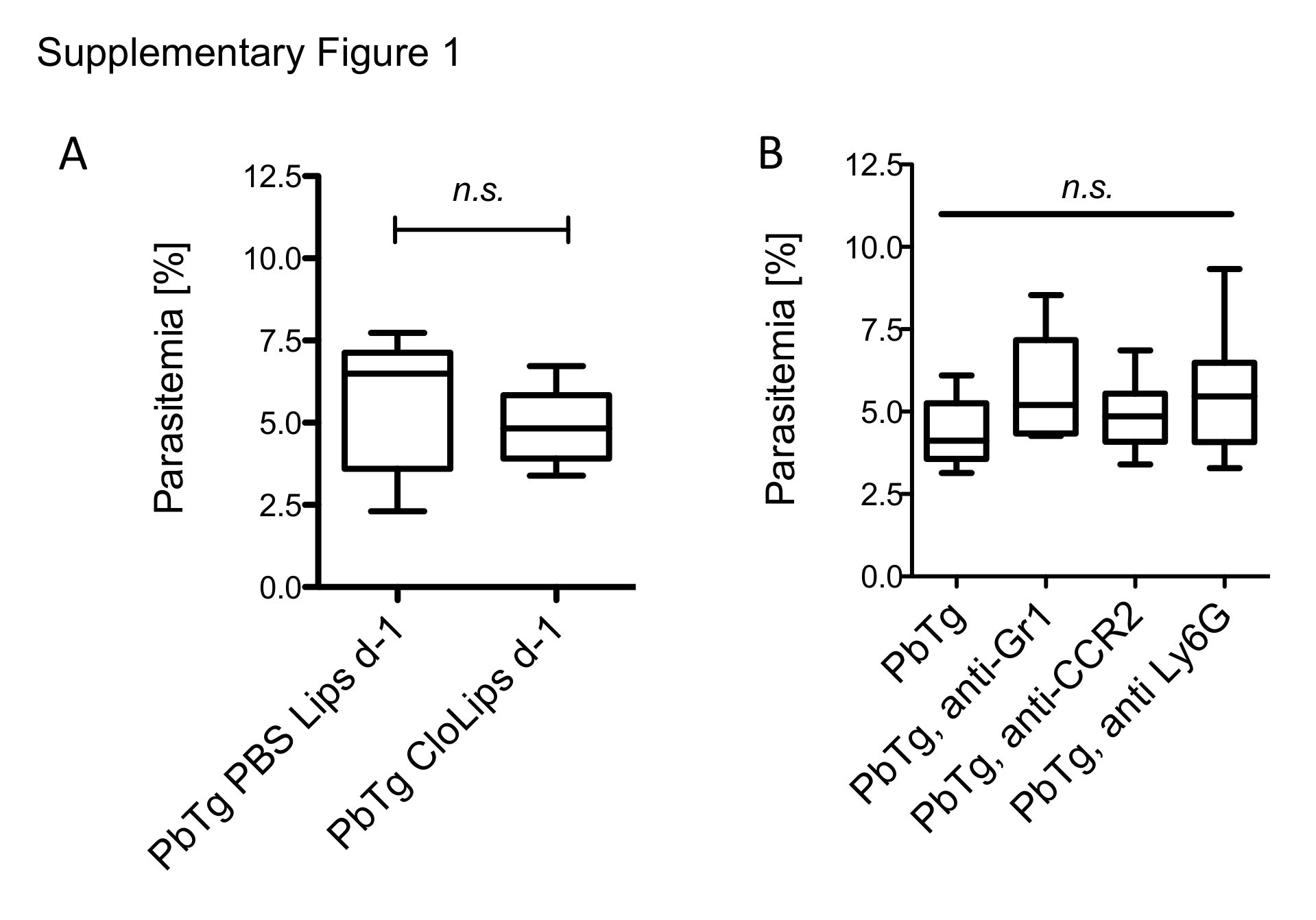

Supplement: S1 Fig — Determination of parasitemia levels on day 6 after infection in the blood of mice that had received either (A) Clodronate liposomes or PBS-filled liposomes one day before infection with 5*105 PbTg-iRBCs or (B) mAb-injected mice on day 6 after infection with 5*104 PbTg-iRBCs. The indicated antibodies were injected on the day of infection as described in the main text. N = 6–8 per group, Statistical analysis was performed by student’s t test (A) or Kruskal-Wallis test (B). (TIF) [file pone.0124080.s001.tif]

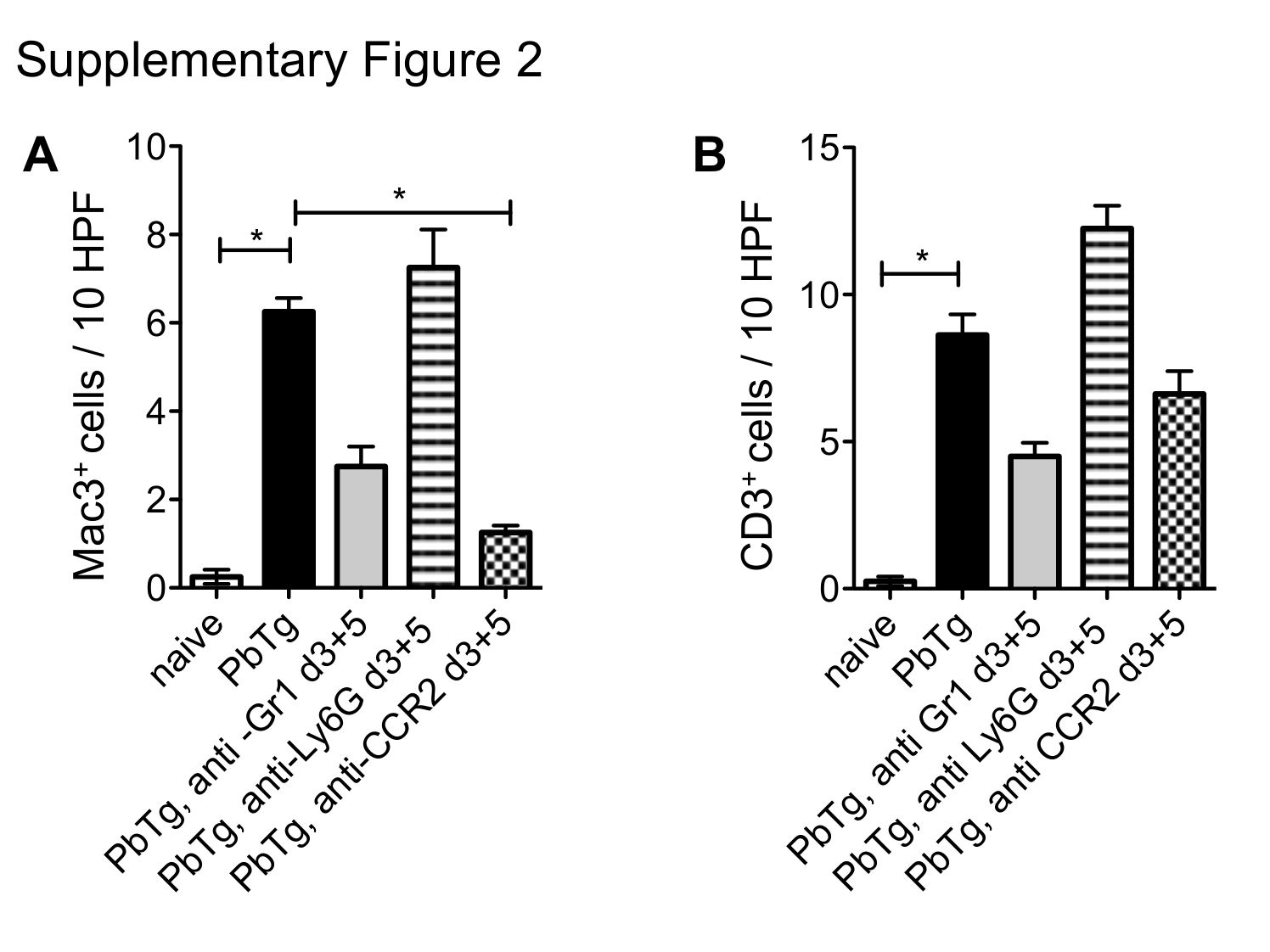

Supplement: S2 Fig — C57BL/6 mice were infected i.v. with 5*10e4 PbTg-iRBC and then subdivided into groups that received either anti-Gr1, anti-Ly6G or anti-CCR2 mAb on days 3 and day 5 p.i. (late depletion). On day 6 p.i., tissue sections from the brains of individual mice were assessed for pathological changes. Quantification of Mac3+ cells (A) and CD3+ T cells (B) in brain tissue sections of individual mice. Bars show mean ± SEM from n = 8 mice per group. Statistical analysis was performed using Kruskal-Wallis test and Dunn’s Post test and significant differences are indicated by the stars in brackets between the groups (* p<0.05). HPF, High Power Field. (TIF) [file pone.0124080.s002.tif]

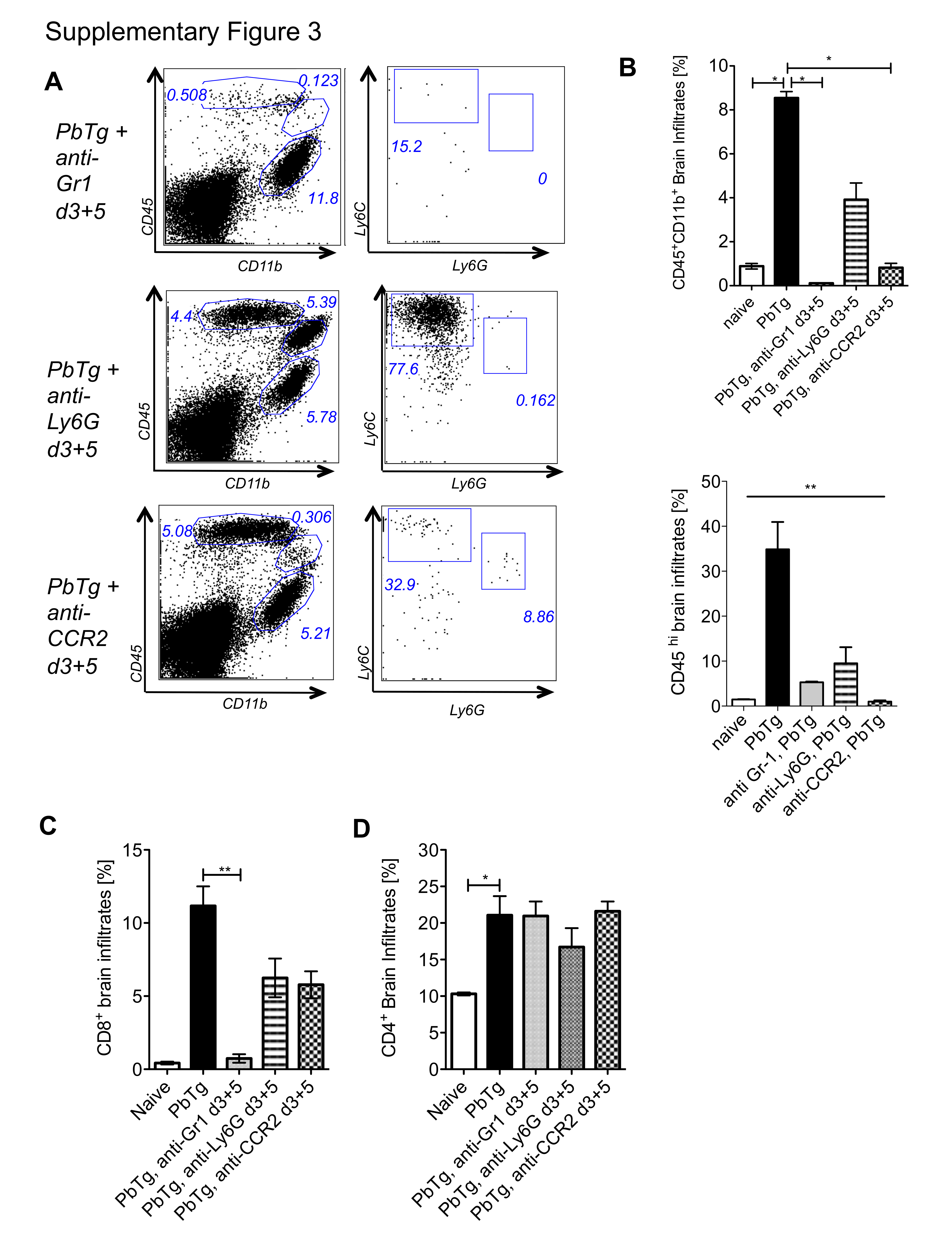

Supplement: S3 Fig — C57BL/6 mice were left either untreated or infected with 5*10e4 PbTg iRBC (see main Fig 4A). (A) In addition, groups of infected mice were treated either with anti-Gr1 (upper plots), anti-Ly6G (middle plots) or anti-CCR2 mAb (lower plots) on day 3 and 5 during PbTg-infection. On day 6 p.i., cellular infiltrates from the brains of individual mice were prepared and analysed for the frequency of infiltrating lymphocytes (CD45hiCD11b-) and mononuclear cells CD45+CD11b+ cells and therein the amount of recruited monocytes (Ly6C+) and neutrophils (Ly6G+) by flow cytometry. Representative plots from one out of four mice are shown. (B) Frequency of CD11b+CD45+ cells (upper graph) and CD45hiCD11b- cells among the brain infiltrates (lower graph). (C, D) CD45+CD11b- cells were then assessed for the expression of CD8 and CD4. Bars show mean ± SEM from n = 4–5 mice per group. Statistical analysis was performed using Kruskal-Wallis test and Dunn’s Post test and significant differences are indicated by the stars in brackets between the groups (* p<0.05). (TIF) [file pone.0124080.s003.tif]

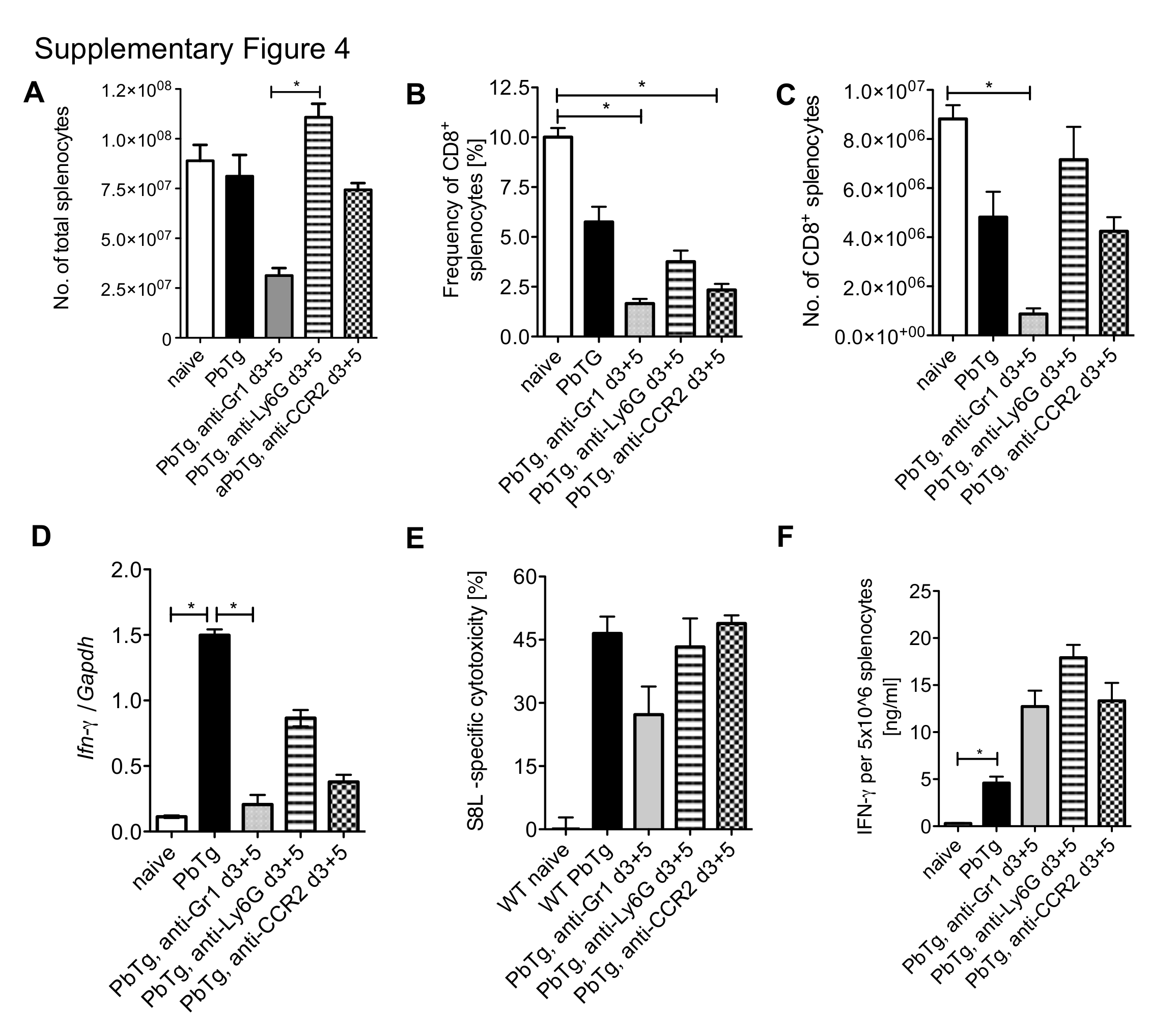

Supplement: S4 Fig — C57BL/6 mice were left either untreated or infected with 5*10e4 PbTg iRBC. In addition, groups of infected mice were treated either with anti-Gr1, anti-Ly6G or anti-CCR2 mAb (on day 3 and 5 during PbTg-infection. (A) Total cell count of splenocytes from all d3+5 depletion groups and controls at day 6 p.i. (B) Frequency of CD8+ splenocytes in percent from all d3+5 depletion groups and controls at day 6 p.i. (C) Calculated total amount of CD8+ splenocytes according to data from B and C. (D) Fold increase of IFN-γ mRNA levels relative to GAPDH in the brains of PbTg-infected mice ± d3+5 mAb depletion on day 6 p.i. n = 6–8 per group, Kruskal Wallis test with Dunn’s Post test was performed. (E) In vivo cytotoxicity assay analysing PbTg-specific T cells at day 6 in the spleens, using SIINFEKL loaded target cells which were adoptively transferred into infected and non-infected mice 18 hours before analysis. (F) Splenocytes from the same animals as in E were re-stimulated with SIINFEKL ex vivo for 24 hours and IFN-γ production was quantified by sandwich ELISA.(A-F) Bars show mean ± SEM from n = 4–5 mice per group. Statistical analysis was performed using Kruskal-Wallis test and Dunn’s Post test and significant differences are indicated by the stars in brackets between the groups (* p<0.05). (TIF) [file pone.0124080.s004.tif]

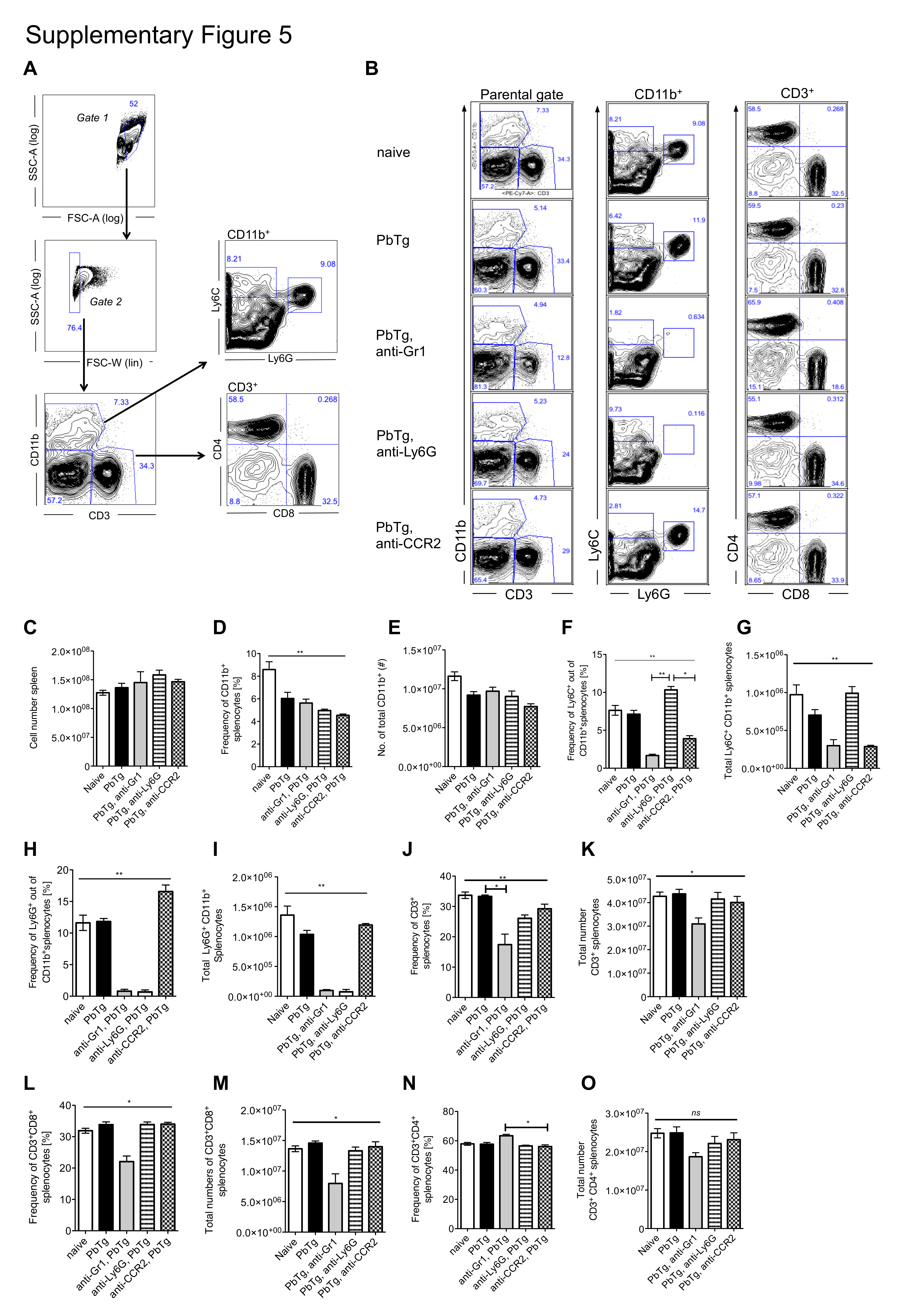

Supplement: S5 Fig — C57BL/6 mice were left either untreated or infected with 5*10e4 PbTg iRBC. In addition, groups of infected mice were treated either with anti-Gr1, anti-Ly6G or anti-CCR2 mAb on the day of PbTg-infection. Two days later, mice were sacrificed for analysis. (A) The diagram illustrates on the left panel the gating strategy for leukocytes from spleen and blood used in flow cytometric analysis. The right panel shows further analysis of CD11b+ gated splenocytes for expression of Ly6C and Ly6G to identify monocytes and neutrophils, respectively, as well as further analysis of CD3+ gated cells for expression of CD4 and CD8. The data show splenocytes from a naïve C57BL/6 mouse. (B) According to the gating scheme shown in (A), splenocytes from all experimental groups were analyzed for the expression of CD3 versus CD11b (left panel). Ly6C+ monocytes and Ly6G+ neutrophils among the previously gated CD11b+ cells are shown in the middle panel, whereas CD3+ gated cells were further analyzed for the expression of CD4 and CD8 (right panel). Representative data of each experimental group are shown, one out of four mice. (C) Total splenocytes count (D-O) Flow cytometric analysis of frequencies and calculation of total amounts of splenic subpopulations, which were gated according to the scheme shown in (A). (D) Frequency and (E) total amount of CD11b+ splenocytes; (F) Frequency and (G) total amount of Ly6C+ monocytes gated from CD11b+ splenocytes; (H) Frequency and (I) total amount of Ly6G+ neutrophils gated from CD11b+ splenocytes; (J) Frequency and (K) total amount of CD3+ splenocytes; (L) Frequency and (M) total amount of CD8+ T cells gated from CD3+ splenocytes, (N) Frequency and (O) total amount of CD4+ T cells gated from CD3+ splenocytes. N = 4 per group, Statistical analysis was performed by Kruskal-Wallis test with Dunn’s Post test. Significant differences are indicated by the stars in brackets between the groups (** p<0.01). (TIF) [file pone.0124080.s005.tif]
